# Development of Geopolymers Based on Fly Ashes from Different Combustion Processes

**DOI:** 10.3390/polym14101954

**Published:** 2022-05-11

**Authors:** Kinga Pławecka, Patrycja Bazan, Wei-Ting Lin, Kinga Korniejenko, Maciej Sitarz, Marek Nykiel

**Affiliations:** 1 Faculty of Material Engineering and Physics, Institute of Material Engineering, Cracow University of Technology, Jana Pawła II 37, 31-864 Cracow, Poland; kinga.plawecka1@pk.edu.pl (K.P.); marek.nykiel@pk.edu.pl (M.N.); 2 Department of Civil Engineering, National Ilan University, No. 1, Sec. 1, Shennong Rd., Yilan 260, Taiwan; wtlin@niu.edu.tw; 3 Faculty of Materials Science and Ceramics, AGH University of Science and Technology, Mickiewicza 30, 30-059 Cracow, Poland; msitarz@agh.edu.pl

**Keywords:** fly ashes, waste incineration product, geopolymers, X-ray fluorescence, X-ray diffraction, mechanical strength

## Abstract

The main aim of this research is to assess different fly ashes as raw materials for the manufacturing of geopolymers. Three different fly ashes have been investigated. First, a conventional fly ash from the Skawina coal power plant (Poland), obtained at a temperature of 900–1100 °C. Second, ultra-fine fly ash from a power plant in China; the side product received at 1300 °C. The third fly ash was waste was obtained after combustion in incineration plants. To predict the properties and suitability of materials in the geopolymerization process, methods based on X-ray analysis were used. The applied precursors were tested for elemental and chemical compounds. The investigations of geopolymer materials based on these three fly ashes are also presented. The materials produced on the basis of applied precursors were subjected to strength evaluation. The following research methods were applied for this study: density, X-ray fluorescence (XRF), X-ray diffraction analysis (XRD), Scanning Electron Microscopy (SEM), flexural and compressive strength. The obtained results show that materials based on fly ashes had a similar compressive strength (about 60 MPa), while significant differences were observed during the bending test from 0.1 to 5.3 MPa. Ultra-fine fly ash had a lower flexural strength compared to conventional fly ash. This study revealed the need for process optimization for materials based on a precursor from a waste incineration plant.

## 1. Introduction

Annual cement production in 2020 was predicted to reach nearly 6 billion tonnes, resulting in a production of approximately 4.8 billion tons of CO_2_. Portland cement productions emits about 7% of global CO_2_. Because of that, alternatives to Portland cement are increasingly being sought [1,2,3,4]. Examples of such alternatives are alkali-activated binders or geopolymers. These materials are formed by the polycondensation of aluminosilicates (containing silicates with alkalis) and are characterized by an amorphous or semicrystalline structure [5,6]. The structural network of geopolymers is based on aluminosilicates, which form a combination of [SiO_4_]^4−^ and [AlO_4_]^5−^ tetrahedra [7,8,9]. These are interconversively linked by oxygen atoms. Bonding usually occurs in a strongly alkaline aqueous solute, but reactivity is also possible in acids, in which the reactive alumino-silicates are dissolved, and then in the polycondensation process the tetrahedrons [SiO_4_]^4−^, [AlO_4_]^5−^ connect at the corners [10,11,12].

**Figure 1:**
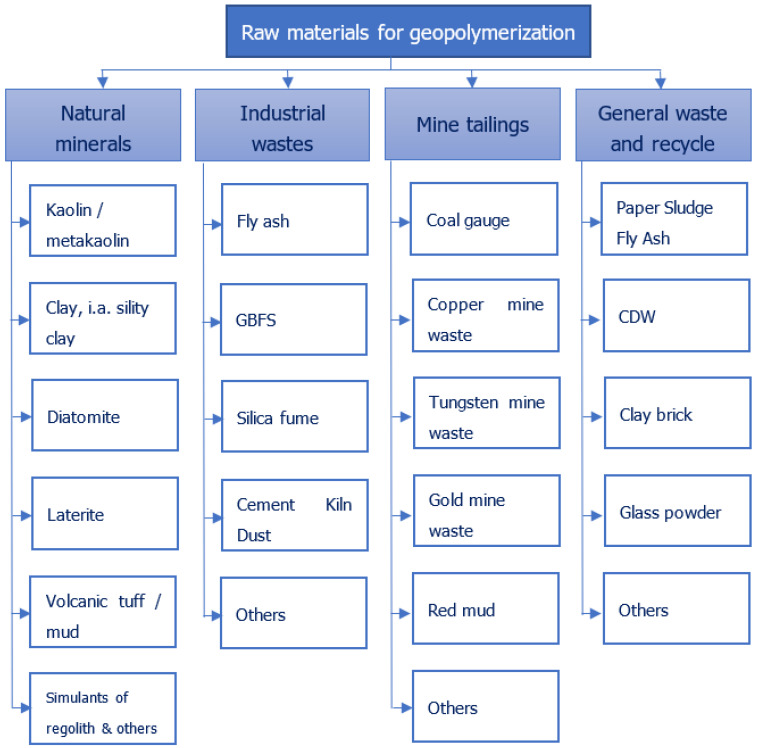
Raw materials used in geopolymerization as aluminiosilicate sources.

Currently, the raw materials most widely applied in geopolymers are metakaolin [13,14,15], fly ash [16,17,18], and slag [19,20,21,22,23]. However, other waste materials can also be used (Figure 1).

Research on geopolymers based on various wastes showed that the geopolymer material exhibits superior strength, corrosion resistance, flame retardancy, and durability [24,25,26,27]. These features give geopolymer a great potential as an environmentally friendly application in construction, but also as a neutralizing material [28,29,30,31,32,33].

All raw materials have their advantages and disadvantages. For example, metakaolin is widely accessible and well known as a precursor for geopolymers. It has repetitive properties (if comes from one source) and is easy to pigment, but its production requires the calcination process of kaolin in high temperature. So, it is energy consuming. Investigations on metakaolin-based geopolymers were carried out using various experimental techniques. The research confirmed that the mechanical strength of metakaolin-based geopolymers was related to the chemical composition, but the porosity of the material also played an extremely important role in the resistance to degradation mechanisms, which is correlated with long-term mechanical properties. Additionally, the microstructure depends on the content of silica, and it affects the mechanical properties of the material. It was found that the greater the compactness of the silica, the higher the strength of the material, and the nature of the structure is uniform and less rough [15,34,35,36].

Fly ashes and slags have an advantage as ready waste from energy processes. These raw materials do not require an additional process to be reactive during geopolymerization, but at the same time these materials are not repetitive, because their composition and properties are connected with used technology of burning, used feed-stock, and other variables. The favorable properties of these materials are connected with the use of energy industry byproduct as well as the fact that these materials do not require additional pretreatment.

Olivia and Nikraz [37] tested a geopolymer based on mixtures of fly ash with the Taguchi method and performed strength tests taking into account the influence of aggregate content, the ratio of alkaline solution to fly ash, the ratio of sodium silicate to sodium hydroxide, and the hardening method [37]. The test results were compared with Portland cement. A recent study showed that fly ash geopolymers had a compressive strength of approximately 55 MPa, which is comparable to cement, and also had a higher tensile and bending strength, but with a lower modulus of elasticity. Research indicated that geopolymers can be an alternative to Portland cement, and at the same time limit the pollution of the environment [37,38].

Geopolymers obtained from fly ash and ground bottom ash were compared. Among others, sodium hydroxide (NaOH) solutions were used as activators. The results indicated that either fly ash or furnace ash could be used as source material to produce geopolymers. The qualities of the geopolymers depended on the precursors used and the concentration of NaOH. Fly ash had more reactivity and resulted in a higher degree of geopolymerization compared to bottom ash. The study also determined the optimal concentration of NaOH activator at the level of 10 M. The strength results showed the advantage of fly ash over bottom ash, achieving a compressive strength of 35 MPa [39].

Kong et al. [39] examined geopolymers made based on fly ash and metakaolin. Both types of geopolymers were produced using sodium silicate and potassium hydroxide solutions. The study of morphology revealed that fly ash-based geopolymers were characterized by smaller porous structures, resulting in lower internal stress, and provided higher-strength properties [39].

In recent years, studies have been conducted on the neutralization of toxic and unfriendly waste as a precursor for the production of geopolymers.

Lo et al. [40] conducted a study to investigate the effect of the partial substitution of traditional Portland cement with municipal waste incineration ash and rice husk ash. The results showed that despite their lower reactivity, the waste materials can be successfully used in building materials [40]. A few studies have examined the use of waste from coal incineration plants, municipal waste, oil-contaminated sand, or the burning of agricultural products in building materials. These studies were conducted to relieve the burden on the environment. Another advantage is the reduction of the amount of base material because these materials can be used as fillers as long as they do not have negative effects on the properties of the base material [41,42,43,44,45].

Lach et al. [46] investigated the possibility of immobilizing waste from municipal waste incinerators in geopolymer materials. The results of geopolymerization presented high immobilization levels of compounds and elements, such as chlorides, sulfates, fluorides, barium, and zinc, and the produced materials had good listing properties [46].

The authors present a comparison of geopolymers made on various fly ashes. Research on conventional fly ash has shown great potential for the use of this material as a precursor in geopolymers not only in the traditional casting method, but also in 3D printing methods, which only confirms the need for more research on geopolymeric materials [47], while the use of fly ash with a larger surface spread, such as ultra-fine reactive fly ash, is becoming more and more popular. The comparison of three different raw materials allowed us to indicate the advantages and disadvantages of the selection for the production of fly ash-based geopolymer materials and their influence on the final product. This research included the analysis of the chemical and phase composition, strength tests, and the analysis of the morphology of the manufactured materials. The article also presents efforts to adapt waste ash after the incineration of municipal waste, which is of increasing interest because of the benefits resulting from immobilization and waste that threaten the natural environment. This kind of investigation is important to provide new knowledge about the possibility to use local resources for manufacturing construction materials by geopolymerization, including information about the most important investigation of raw materials before classification as a possible material for the geopolymerization process. A comparative analysis for these particular fly ashes has not been provided before in the literature.

## 2. Materials and Methods

### 2.1. Materials and Samples Preparation

In this work, attempts were made to produce geopolymers based on three types of fly ashes: two types of fly ash from power plants and one waste obtained after the incineration of municipal waste. Conventional class F fly ash was obtained from Skawina Heat and Power Plant (Skawina, Poland). The size distribution and spherical shape of the fly ash particles in conventional fly ash as well as the crystalline internal structure result in a material with good workability. Another precursor used to make the geopolymer was Ultra-Fine Reactive Fly Ash (Rufa), from a power plant in China (TRIAXIS Corporation, Hong-Kong, China). Conventional fly ash is a product of a thermal power plant heated to 900–1100 °C, and RUFA is a product of a thermal power plant heated to 1300 °C. The high temperature causes the cracking of the ash microspheres at about 1100 °C and completely collapses at about 1300 °C in the particle separation process. The resulting particles have a much larger specific surface. Although the chemical composition is comparable to that of conventional fly ash, RUFA can compete successfully with conventional fly ash in terms of pozzolanic activity [48]. The last type of precursor was waste obtained from municipal waste incineration (Białystok, Poland). The tested fly ashes were not subjected to any pretreatment.

**Figure 2:**
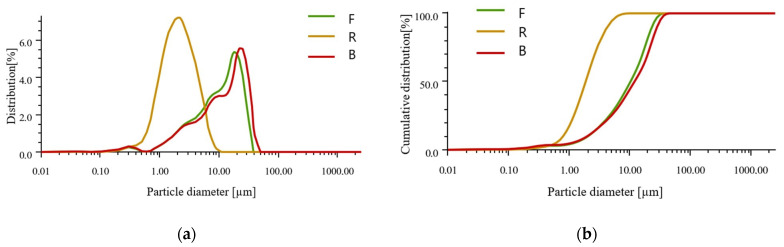
Results of particle size analysis: (**a**) Particle size distribution histogram; (**b**) cumulative particle size distribution curves.

Figure 2 and Table 1 show the histogram of the particle size distribution and the cumulative particle size distribution curves according to the percentage of particles for applied fly ashes. The research was carried out using a Particle Size Analyzer (AntonPaar GmbH, Graz, Austria).

The particle size distribution in all types has the Gaussian plot character. This is most visible for the R fly ash. The characteristics for fly ash F and Fly ash B form waste obtained after incineration of municipal waste are similar. The mean size of the particles is lower for fly ash (ultra-fine) and it is about 2.5 µm, whereas for fly ash F it is approximately 12 µm and for B it is approximately 14.5 µm, respectively.

The alkaline activator was a 10 M sodium hydroxide solution (PCC Rokita SA, Brzeg Dolny, Poland), and sodium water glass R-145 (STANLAB, Gliwice, Poland) with a molar module of 2.5 and a density of about 1.45 g/cm^3^; Na/Al ratio was 1:2. To prepare the mass, the precursors were mixed with the activator for about 10 min and poured into the molds. The molds were placed on a vibrating table to remove air bubbles. After preparing the masses, the samples tightly covered with foil were placed in a laboratory drier (SLW 750 STD, Pol-Eko-Aparatura, Wodzisław Śląski, Poland) for 24 h at a temperature of 75 °C. Loss of material mass after 24 h of heating in the furnace was less than 0.1%. The samples were unmolded and cured in laboratory conditions (temperature ca. 20 °C, relative humidity ca. 50%) for 28 days. As a next step, the strength tests were performed. Table 2. lists the names of the samples for better systematization and mixing proportion.

### 2.2. Research Methods

#### 2.2.1. Density

The actual density of the applied precursors and the density of the manufactured geopolymer were determined. The actual density was determined with a Pycnomatic ATC (Thermo Fisher Scientific, Massachusetts, USA) with the PN-EN ISO 18753: 2006 standard (“High-quality ceramics (advanced ceramics, technical advanced ceramics)—Determination of the actual density of ceramic powders using the pycnometric method”). Pycnometers use a gas displacement technique to determine the actual density. Helium was used as the gas because helium atoms have very small diameters and can penetrate even extremely small pores in solids. As the outer surface of the samples did not show significant roughness, the density of the samples was determined using the geometric method for solid materials. The density was determined as the mean of the measurements for the four samples. The samples were measured with a laboratory caliper with a measurement accuracy of 0.01 mm, and the mass of the samples was determined with a laboratory precision analytical balance RADWAG PS 200/2000.R2 (maximum load: 200/2000 g; reading accuracy: 0.001/0.01 g).

#### 2.2.2. Chemical Composition of Precursors

The chemical and mineral composition was analyzed with the use of spectroscopy (X-ray diffraction and X-ray fluorescence). X-ray fluorescence (XRF) was conducted on a WDXRF AxiosmAX Spectrometer equipped with an Rh 4 kW source (PANalytical, Malvern, UK). X-ray diffraction (XRD) was evaluated with an X’Pert Pro MPD diffractometer (PANalytical, Malvern, UK) with CuKα radiation at 30 mA and 40 kV. The 2θ angle was varied from 20 ° to 53 ° with a step of 0.04 ° and an accumulation time of 7 s for each step. XRD was conducted to assess the mineral composition of materials from various waste streams used in the research. The calculated values of the distance between the planes were used to identify the phases contained in the tested materials. X-ray analysis was performed using HighScore Plus software. To analyze the presence of phases, the PDF4 + crystallographic database was used.

#### 2.2.3. Strength Tests

Compressive strength tests were performed following the EN 12390-3 standard (“Tests for hardened concrete. Compressive strength of samples”), on cubic samples (50 mm × 50 mm × 50 mm), using the Matest 3000 kN universal testing machine (Matest, Treviolo, Italy). The bending strength tests were executed following the EN 12390-5 standard (“Tests of hardened concrete. Bending strength of test specimens”) using the same Matest 3000 kN testing machine. Sample dimensions were 50 mm × 50 mm × 200 mm. The distance between the support bars was 150 mm. The test speed was set up to 0.05 MPa/s. The strength tests were performed after 28 days of sample conditioning. The minimum number of samples was five, and the values reported in the results are average values.

#### 2.2.4. Microstructure

The geopolymers produced were subjected to microscopic observations to define the formed structure. Microscopic observation was performed using a JEOL JSN5510LV Scanning Electron Microscope (JEOL Ltd., Tokyo, Japan). The samples after mechanical properties research were used for this test. Before testing, the surface of the sample was covered with a conductive gold layer on the JOEL JEE-4X vacuum evaporator (JEOL Ltd., Tokyo, Japan).

## 3. Results and Discussion

### 3.1. Density Results

**Figure 3:**
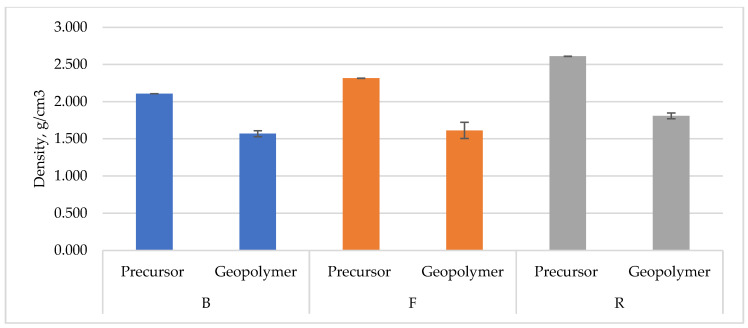
Density results of tested materials.

The first stage of the studies was to determine the density of the precursor used and the density of the produced geopolymers. Figure 3 shows a comparison of the actual density with the density determined after the geopolymerization process by a specific geometric method. The actual density of fly ashes was at a similar level and reached 2.315 g/cm^3^ for conventional fly ash (F) and 2.610 g/cm^3^ for ultra-fine reactive fly ash (R). The actual density of the material obtained after the municipal waste utilization (B) was equal to 2.107 g/cm^3^. These values are in line with the properties given for bulk geopolymeric solid materials in the literature [4,46].

An important element of the density tests is the comparison of the density of the precursor with the results obtained after the geopolymerization process. That gives an initial view of the porosity of the manufactured samples. The largest differences were noticed for sample B. However, large differences also occurred in the case of geopolymers made using fly ashes, which suggests the relatively high porosity of the tested materials. It is also worth noting that standard deviation given for these samples is relatively small, as the samples are repetitive.

The presence of porosity in geopolymer concrete is related to inaccurate compaction of the material. It has been proven that a porous structure can have a negative effect on strength properties [49,50]. The research provided by Mindess [51] shows that the presence of pores, pore size, and pore distribution significantly affected compressive strength by causing a decrease in compressive properties as the proportion of large pores in the material structure increased. While another reason was the phase composition of the samples [51]. Luna-Galiano et al. [52] indicated that sodium hydroxide-activated geopolymers had higher porosity compared to potassium hydroxide-activated geopolymer materials. In addition, they also indicated that there were differences between the activation of sodium hydroxides and silicates, where the materials containing the sodium activator were represented by a higher proportion of porosity and the pores had a larger diameter. In addition, the activator used and the curing temperature greatly affect the properties of geopolymers [52]. They revealed that the structure of geopolymers activated with potassium hydroxides or potassium and sodium hydroxides contained a higher proportion of porosity compared to material activated with sodium hydroxide. However, the authors did not indicate an explanation for this phenomenon [53]. Kong et al. [39] investigated the effect of elevated temperature on geopolymer materials. According to their results, the porosity of geopolymers depends on thermal activation during curing. Elevated temperature causes a decrease in pore size. However, the volume proportion of the volume of pores increases compared to the material cured at room temperature [39]. Other researchers confirmed this effect, finding that high-temperature curing produced porosities within 0.01 µm, while geopolymers cured at room temperature contained pores with diameters in the range of 0.1–1 µm [54].

### 3.2. Chemical Compositions Results

The chemical composition of the tested precursors was determined by the XRF method. The results are presented in Table 3. Fly ash mainly contains oxygen, silicon, calcium, aluminum, iron, potassium, sodium, magnesium, titanium, and phosphorus. Conventional fly ash (F) contained a higher aluminum content and ultra-fine reactive fly ash (R) exhibited a higher calcium content. Analysis of the chemical composition of the ash obtained from the municipal waste incineration plants (B) showed that the ash consisted mainly of calcium, chlorine, oxygen, sulfur, potassium, sodium, and zinc. According the ASTM-C618-2, based on oxygen composition, the fly ashes that came from combustion process can be classified as a class F and fly ash form the municipal waste incineration plants is in class C. Note that for the formation of bonds of geopolymers, the oxygen composition of the base material is important. A large amount of calcium could cause too rapid a reaction of the materials, which would not allow to form the 3D structure that is characteristic for geopolymers [55].

Table 3 shows the oxide composition of the materials tested. Fly ash (F) was characterized by the highest content of aluminum and silicon oxides, which are necessary components for the geopolymerization process to take place. Additionally, for all these fly ashes, the concentrations of potentially hazardous elements, such as Ce, Y, and Sr, are relatively low and do not cross the standards for non-hazardous wastes. Because of this, using these fly ashes as a raw material for building products is possible from a legal point of view [46,55].

Previous research showed that high calcium fly ash is suitable as a source material for making good quality geopolymer materials [56,57]. Calcium in fly ash results in the formation of a calcium silicate hydrate (C-S-H) phase. The coexistence of this phase with the geopolymer gel has a positive effect on the strength properties of the obtained geopolymer materials. Moreover, high calcium content results in the fact that calcium atoms bond in the geopolymer network and act as charge-balancing cations [58].

Table 4 presents a summary of the identified phases for the diffractogram recorded for sample B.

Sample B was characterized by a high content of calcite and chlorocalcite. Additionally, anhydrite, quartz, and kaolinite were also identified.

Table 5 presents a summary of the identified phases for the diffractogram recorded for sample F.

**Figure 4:**
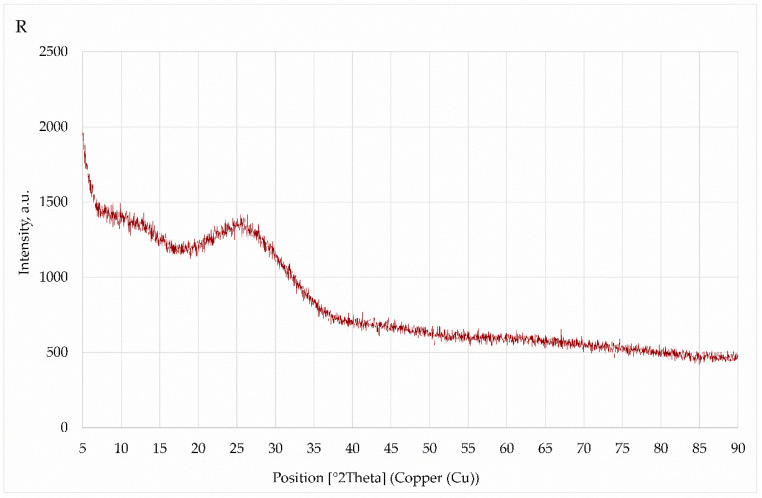
Diffractogram for ultra-fine fly ash R.

In terms of the investigation of the diffraction patterns, the conventional fly ash was characterized by a high quartz content, mullite, hematite, and alite. Figure 4 shows the diffractogram recorded for ultra-fine fly ash (R) obtained from China. The material was almost completely amorphous. The raised background at angles of 20–30 2θ angles is the so-called amorphous halo.

The results of the X-ray studies revealed that the presence of characteristic crystalline phases, such as quartz, mullite, anhydrite, or hematite, could be detected in the ashes analyzed. They do not participate directly in the polymerization process. In the recorded spectrum for ultrafine fly ash, a large hump can be observed, which indicates the presence of an amorphous phase. The amorphous phases play an important role in the geopolymerization process, and this phase participates in the polymerization reaction [59,60,61,62].

### 3.3. Mechanical Properties and Structure Observations

The initial raw materials for geopolymers strongly influence the resulting microstructure, although structures may be found to be similar due to the presence of the same bond between silicon and aluminum atoms and the presence of a gel phase binder [28]. Strength tests, such as compressive strength, are the basis for evaluating the correctness of the geopolymerization process, as well as for evaluating the suitability of the produced [63]. The compressive strength of geopolymer materials is dependent on several factors, such as the structure, the presence of a crystalline phase, the content and strength of the gel phase, the arrangement and toughness of the insoluble Al-Si particles, and the surface reaction between the gel phase and the insoluble Al-Si particles [64]. Furthermore, variables, such as % CaO, % K_2_O, and alkali type, have a strong correlation with compressive strength. The significance of the Si/Al molar ratio for the alkali dissolution of particular minerals indicates that compressive strength is obtained through complex interactions between the mineral surface, kaolinite, and concentrated sodium silicate solution. After the geopolymerization process, the insoluble particles remain cemented into the matrix, so that the toughness of the mineral is positively correlated with the residual compressive strength [65].

**Figure 5:**
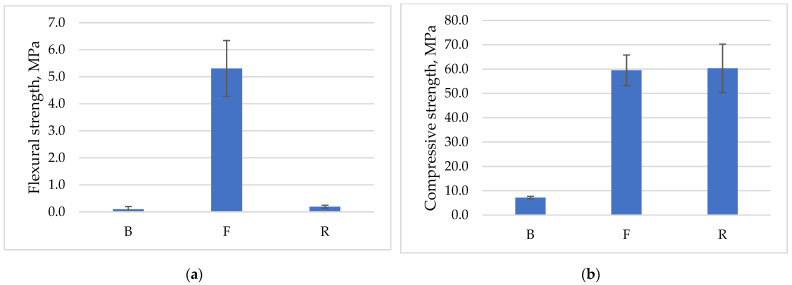
Results of mechanical properties tests: (**a**) Flexural strength; (**b**) Compressive strength.

Figure 5 presents a summary of the strength tests performed for each geopolymer material. The highest value of flexural strength was obtained for the sample based on conventional fly ash from (F), 5.3 MPa. The lowest value was obtained for the material based on ash from the waste incineration plant in Białystok (0.1 MPa), B. A marginally higher value was achieved for the geopolymer based on ultra-fine fly ash (R), but the flexural strength was extremely low and reached about one-twentieth of the geopolymer based on conventional fly ash (Figure 5a). The obtained results are correlated with the high calcium content (more than 30%) and a marginal Al and Si content of about 1%, which, as already mentioned, are extremely important in the geopolymerization process. Ban et al. [66] studied geopolymer mortars based on fly ash, which was replaced by wood ash; it was characterized by high calcium content. The flexural strength of geopolymer mortars, in this case, decreased as the proportion of wood ash in the mix increased, regardless of the choice of curing method [66].

The great difference in the compression test results can be seen in Figure 5b. The fly ash-based geopolymers had comparable compressive strengths and oscillated around 60 MPa. The weakest compression test results were obtained for the material based on ash from the incinerator plant, sample B, and were approximately 7 MPa, which is related to the chemical composition and high porosity of this material.

Mechanical property tests revealed the strengths and weaknesses of the materials studied. The geopolymers produced on the basis of conventional fly ash exhibit high strength properties in both compression and flexural strengths. The material based on ultra-fine fly ash displayed good compressive strength. However, in the case of point load application, it is a material that requires reinforcement, e.g., long fibers, which could reduce such rapid cracking of this material. In the case of ash obtained after the incineration of municipal waste, it must be stated that despite the bonding between the components, this material does not meet even the lowest strength requirements and is not suitable for use in this way. The solution to this problem may be optimization of the activator content, which in this case seems to be too large and causes some process of foaming, reducing the strength properties or utilization of this type of waste by adding small fractions of the waste to materials based on other fly ashes [66,67].

### 3.4. Microscopic Observations

Scanning electron microscopy (SEM) enables the visual examination of material in the millimeter to micrometer range to obtain conclusive topographical information and allows the evaluation of structures that cannot be revealed by other methods [68].

**Figure 6:**
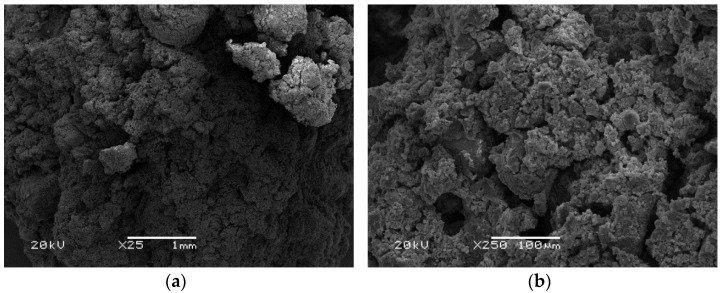

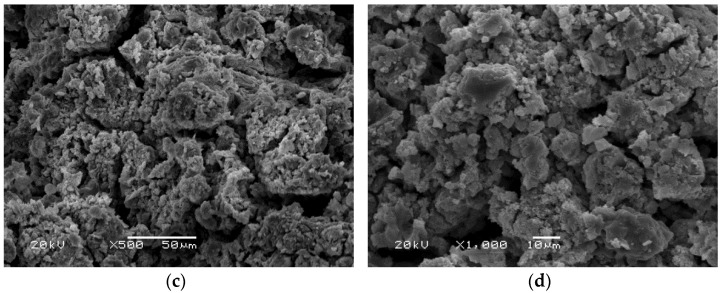
SEM pictures of materials based on waste incineration product in magnification: (**a**) 25×; (**b**) 250×; (**c**) 500×; (**d**) 1000×.

**Figure 7:**
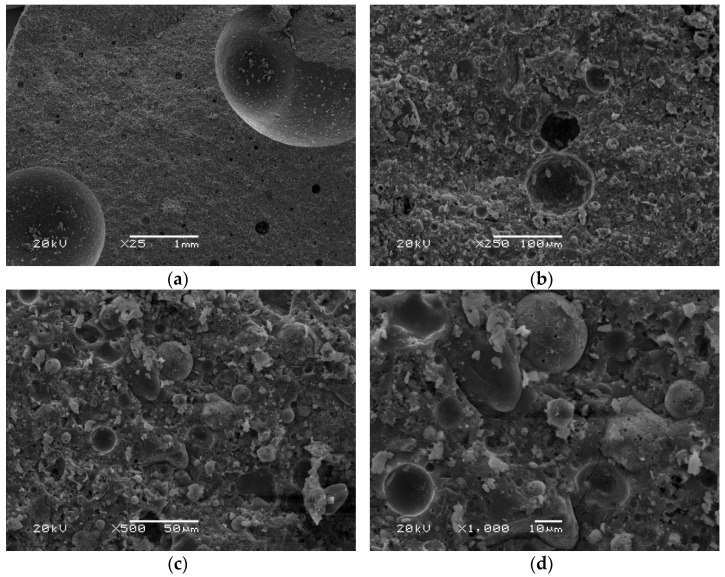
SEM pictures of materials based on conventional fly ash obtained from Skawina Power Plant (F) in magnification: (**a**) 25×; (**b**) 250×; (**c**) 500×; (**d**) 1000×.

**Figure 8:**
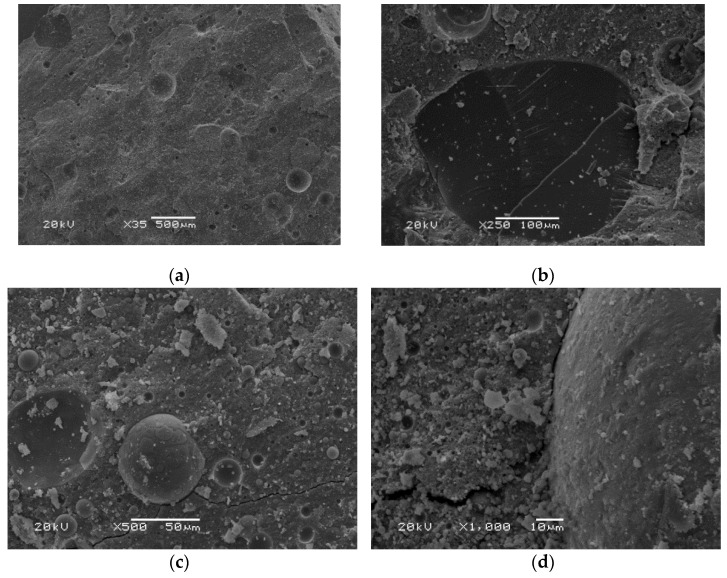
SEM pictures of materials based on ultra-fine fly ash—RUFA (R) in magnification: (**a**) 35×; (**b**) 250×; (**c**) 500×; (**d**) 1000×.

Figure 6, Figure 7 and Figure 8 show the microstructure of the tested materials. In Figure 6, microscopic photos of materials produced based on ash from a municipal waste disposal plant are presented. An inconsistent structure of the feathery material can be observed. At higher magnifications, a highly porous structure was observed, and the pores were polygonal. The structure of the material was brittle and segmented, resulting in very low strength properties. Figure 7 presents photos of geopolymers made using conventional fly ash. A compact structure of the material with visible pores with a maximum size of about 1.5 mm can be observed. However, the pore size was mostly 10–50 µm. The structure was amorphous. Undiluted ash particles can also be seen. This is a typical structure for geopolymers composed of traditional fly ash [69]. Alehyen et al. [70] investigated and described the microstructure of fly ash-based geopolymer mortars. They characterized it as a porous heterogeneous mixture, where some of the ash grains did not react or partially reacted. Additionally, they noted the presence of residual alkaline deposits and geopolymer gel [70].

Observations of the microstructure of the material made from ultra-fine fly ash, RUFA (Figure 8), showed an equally compact and amorphous structure of the material. Significantly lower roughness was observed in comparison to the material based on conventional fly ash. The pore size was a maximum of about 200 microns and the pores were mostly in the range of 10–20 microns. Cracks on the fracture surface with a length of about 300–600 μm were also noticed. The smaller distribution and pore size in geopolymers based on ultrafine fly ash may be related to the formation of colloids C-S-H. The formed colloids contribute to a reduction in the number of capillary pores. Lin [48] studied ultrafine fly ash added to cementitious materials. The microstructure showed that the fine particles contained silica dust and acted partly as an inert material. This contributed to improving the packing density of the particles in the microstructures of the material [48]. The cause of microcracking was shrinkage during drying, which is a physical property of a gel. This is related to the removal of water during the polycondensation process, which causes capillary tension in gel matrices [70].

The presented microstructure images show that the fly ash-based materials are characterized by amorphous structures and contain undiluted fly ash particles. The variable pore content in the microstructure of the materials was also noticed. When the microstructures of geopolymers based on conventional fly ash and ultrafine reactive fly ash are examined, better compaction of the material and thus better bonding for a material based on RUFA ash can be seen.

Similar structures were concluded by Škvára et al. [54]. This research held that the basic mass of the fly ash-based geopolymer was amorphous glass in which minority needle configurations were rare. They also observed remnants of the original ash parts in the geopolymer mass, where the influence of gradual decomposition was evident. The materials produced by the authors showed relatively high porosity (up to 50%) regardless of the nature of the preparation conditions [54].

## 4. Conclusions

This work included structural and strength studies conducted to analyze and select potential precursors that could form the basis of advanced geopolymer materials. When evaluating the suitability of raw materials for geopolymer materials characterized by their ability to form a cementless bonding binder, many factors must be considered, e.g., the content of active ingredients that transfer into solution under the action of an alkaline activator, the ratio of SiO_2_ to Al_2_O_3_, amorphous phase composition and content, grain size, particle density, and unburned carbon. Many studies have confirmed that fly ash can become a source that can be used in the geopolymerization process, which is also presented in this paper. However, it should be noted that, depending on the combustion temperature, fly ash as waste material is not always a precursor that will provide adequate strength properties. The provided test allows us to formulate following conclusions:

- Mechanical tests showed that materials made from fly ash obtained from the Skawina Power Plant had better strength properties compared to other materials analyzed.
- The ultrafine fly ash, in which the bending strength was low, can be also useful material for geopolymers preparation, but it requires some reinforcement. This kind of reinforcement could be, for example, glass fiber, to improve the bending properties.
- Studies conducted with incineration waste indicate that this specific type of ash is not suitable for the production of alkali-activated materials under the conditions presented. There were not enough strong bonds in this type of material, resulting in an extremely developed material structure that was very brittle and did not meet the minimum strength requirements.

The next stage of the research will be the optimization of the geopolymerization process in terms of activator selection as well as the pre-treatment of precursor from waste incineration because in the presented research this material has not been subjected to such treatment and the research results indicate the need for further research in this direction. Waste immobilization is an extremely important area of research, which was also noted by other scientists. A possible solution could also be to add a small fraction of this material to the geopolymeric material as a method of utilizing this type of waste, which seems possible because of the observed microstructures.

## Figures and Tables

**Table 1:** Particle size distribution in different materials.

Index	D_10_ [µm]	D_50_ [µm]	D_90_ [µm]	Mean Size [µm]
**F**	2.040	10.235	23.167	12.167
**R**	0.7922	1.8683	4.266	2.418
**B**	1.9866	11.924	28.063	14.513

**Table 2:** List of manufactured geopolymers.

Index	Description	Mix Proportion
**B**	Geopolymer based on the precursor of the municipal waste incineration plant (Białystok, Poland)	4 kg of precursor + 10 M sodium hydroxide solution + water glass (1200 mL in total)
**F**	Geopolymer based on the precursor of the Power Plant in Skawina (Skawina, Poland)	
**R**	Geopolymer based on precursor from the Power Plant in China (TRIAXIS Corporation).	

**Table 3:** The analyzed chemical formula of tested fly ashes.

Precursor	F		R		B	
	Compound Formula	Conc, %	Compound Formula	Conc, %	Compound Formula	Conc, %
1	Na_2_O	1.714	Na_2_O	1.950	Na_2_O	3.277
2	MgO	1.636	MgO	1.214	MgO	0.438
3	Al_2_O_3_	25.499	Al_2_O_3_	16.639	Al_2_O_3_	1.273
4	SiO_2_	50.897	SiO_2_	50.914	SiO_2_	3.994
5	P_2_O_5_	0.469	P_2_O_5_	2.013	P_2_O_5_	0.387
6	SO_3_	1.276	SO_3_	0.238	SO_3_	10.672
7	K_2_O	3.007	K_2_O	3.532	K_2_O	4.350
8	CaO	5.306	CaO	15.435	CaO	43.323
9	TiO_2_	1.456	TiO_2_	1.602	TiO_2_	0.719
10	Cr_2_O_3_	0.030	Cr_2_O_3_	0.025	Cr_2_O_3_	0.037
11	MnO	0.111	MnO	0.078	MnO	0.065
12	Fe_2_O_3_	8.001	Fe_2_O_3_	5.509	Fe_2_O_3_	1.060
13	NiO	0.017	Co_3_O_4_	0.015	CuO	0.082
14	CuO	0.024	NiO	0.024	ZnO	2.872
15	ZnO	0.036	CuO	0.054	SrO	0.051
16	Rb_2_O	0.023	ZnO	0.112	ZrO_2_	0.023
17	SrO	0.078	Ga_2_O_3_	0.039	CdO	0.029
18	ZrO_2_	0.043	SeO_2_	0.015	SnO_2_	0.085
19	BaO	0.091	Rb_2_O	0.024	SbO_2_	0.064
20	CeO_2_	0.032	SrO	0.236	BaO	0.073
21	PbO	0.024	Y_2_O_3_	0.015	PbO	0.420
22	Cl	0.230	ZrO_2_	0.096	Cl	26.477
23			BaO	0.048	Br	0.215
24			PbO	0.083	I	0.014
25			Cl	0.088		

**Table 4:** Identified phases and their percentage share in the B -sample.

Sample ID	Identified Phases	Chemical Formula	Amount of Phase
B	Calcite (Calcium Carbonate)	CaCO_3_	43.0
	Chlorocalcite	CaCl_3_K	35.0
	Anhydrite	CaSO_4_	14.4
	Quartz	SiO_2_	7.5
	Kaolinite	Al2Si_2_O_5_(OH)_4_	0.1

**Table 5:** Identified phases and their percentage share in conventional fly ash F.

Sample ID	Identified Phases	Chemical Formula	Amount of Phase
F	Quartz	SiO_2_	47.8
	Mullite	Al_6_Si_2_O_13_	48.4
	Hematite	Fe_2_O_3_	1.6
	Alite	Ca3SiO_5_	2.2

## Data Availability

Not applicable.
